# Natural Rubber/Dendrimer Modified Montmorillonite Nanocomposites: Mechanical and Flame-Retardant Properties

**DOI:** 10.3390/ma11010041

**Published:** 2017-12-28

**Authors:** Chenyang Zhang, Jincheng Wang

**Affiliations:** College of Chemistry and Chemical Engineering, Shanghai University of Engineering Science, Shanghai 201620, China; zcy9372@163.com

**Keywords:** FR-DOMt, natural rubber (NR), nanocomposite, structure, properties

## Abstract

A series of flame-retardant nanocomposites were established based on compounding of natural rubber (NR) and dendrimer modified flame-retardant organic montmorillonite (FR-DOMt). The merits of these nanocomposites were focused on their better mechanical and flame-retardant properties. X-ray diffractometer (XRD) together with scanning electron microscopy (SEM) and transmission electron microscopy (TEM) analysis revealed that exfoliation, intercalation, or aggregation status in the NRmatrix can be achieved by addition of different amounts of FR-DOMt. The sound effects of blend ratio of FR-DOMt on mechanical, thermal stability, and flame-retardant (FR) properties of NR were studied. The NR/FR-DOMt-20 composite possessed the highest tensile strength, and this resulted from complicated interactions between layered silicates and elastomers. In addition, with loading of 20 phr of FR-DOMt, the flammability parameters of NR, such as heat release rate (HRR), smoke evolution area (SEA), and carbon monoxide (CO) concentration, were obviously reduced from cone calorimeter analysis.

## 1. Introduction

In order to reduce polymer consumption and production cost, clay minerals including montmorillonite (Mt), saponite, hectorite, etc., were widely used as fillers in polymeric industry for many years [1]. In the past decades, nanocomposites based on polymer matrix and organoclay were designed and produced with high mechanical properties and thermal stability [2,3,4,5]. Much attention was paid to rubber nanocomposites filled with organoclay recently, which included chloroprene rubber [1], cis-1,4-polybutadiene rubber [6,7], fluoroelastomer [8], ethylene-propylene-diene terpolymer [9,10], nitrile rubber [11], and isobutylene-isoprenerubber [12].

Compared with synthetic rubbers, natural rubber (NR) possessed unique advantages required for manufacturing of aerotires, suspension elements, various latex products, etc. However, NR had the shortcoming of high flammability. This setback may limit its application in mine conveyor belts, power cables, aircraft tire treads, etc. [13].

The compounding of NR matrix with inorganic flame-retardant additives was an effective and economical way to improve its flame-retardant behavior [14,15,16]. Due to small size, high thermal stability, and intercalation abilities, Mt has been paid special attention in the field of flame-retardancy. It was hard to prepare intercalated or exfoliated NR/Mt nanocomposites due to no polar groups in the backbone of NR. Lots of efforts were focused on improving the compatibility between NR and Mt by using organic modification methods. Khanlari et al. [17] studied the thermal stability, flame-retardance together with the hardness and mechanical properties with addition of organoclay in NR nanocomposites. Its aging hardness was decreased more than 55% and the ignition time was delayed about 150% by loading 3 wt % of organoclay in NR. In addition, the decreased peak value of heat release rate was about 54% compared to the pure NR. Liu et al. [18] researched the nanocomposites of NR and tributyl phosphate modified Mt (TMt) by the mixing intercalation method. Their flame-retardant property was tested by cone calorimeter analysis. Results showed that the NR/TMt nanocomposites presented better flame-retardant properties. The heat release rate, mass loss rate, and smoke producing rate of NR/TMt nanocomposites were obviously reduced. Efforts have also been made to combine organoclay and other flame-retardant additives [14,19].

In the near future, more attention was focused on dendrimers, a novel type of polymer which possessed porous networks, reactive end groups, less crystallization ability, and good compatibility with other macromolecules [20]. In addition, dendrimers owned the special features of step by step controlled synthesis and together with their synthesis from monomers, which were resulted from molecular and polymer chemistry-like properties [21,22]. In our previous work, a novel type of flame-retardant dendrimer modified OMt (FR-DOMt) was prepared and characterized [23]. It was disclosed that the employment of dendrimers can increase the d001-value and thermal stability of organoclay. In addition, this dendrimer containing phosphorus and boron elements behaved like a surfactant for surface modification. As can be deduced, the flame-retardance and smoke inhibition effects of phosphorus, boron, and silicon elements in FR-DOMt can be used to fabricate a new type of enhanced clay-polymer nanocomposites (CPN).

In the present work, NR/FR-DOMt nanocomposites were prepared using this novel type of organoclay. The NR nanocomposite with 20 phr of FR-DOMt exhibited better mechanical properties. Moreover, this NR nanocomposite also possessed excellent thermal stability and flame-retardance. 

## 2. Experimental Section

### 2.1. Materials

A type of flame-retardantand dendrimer modified OMt, named as FR-DOMt, was prepared by us [23]. To compare the physical, mechanical, and flame-retardant properties of different NR nanocomposites, following formulation, which was presented in Table 1, was used.

### 2.2. Preparation of FR-DOMt

The water solution of tetra-hydroxymethyl phosphonium chloride (THPC, 8.57 g) was placed in a three-necked flask. Inorganic Mt (40 g) was added into above mixture, and a cation exchange reaction was occurred. THPC-OMt was thus obtained. *N*,*N*-dihydroxyl-3-aminmethyl propionate (27.54 g) and toluene-p-sulfonic acid (0.07 g) were gradually added to above THPC-OMt. The resultant dispersion was vigorously stirred for 10 h. In addition, this reaction was repeated for three times, and the dried cake was ground to obtain different generations of dendrimer modified OMt, DOMt-1,2,3. Last, boric acid (71.23 g) was added into above DOMt-3 in a three-necked flask provided with a stirrer. The mixture was treated at 75~80 °C for about 2 h. The liquid product was distilled, and thus FR-DOMt was obtained [23].

### 2.3. Preparation of NR Nanocomposites

On a double roller plasticator (Shanghai Sinan Rubber Machinery Company, Shanghai, China), NR gums were mixed with different common additives which was illustrated in Table 1. After mixing for about 15 min, FR-DOMt was added in different amounts to the mixture. Thus, different types of NR mixtures were prepared. Before adding into NR matrix, FR-DOMt was dried and cured at 150 °C for about 2 h. Then, the mixtures were placed in a dumbbell mould, and the corresponding curing was carried out at 150 °C for 10 min. Last, different NR nanocomposites were successfully prepared.

### 2.4. Characterization of NR Nanocomposites

X-ray diffraction (XRD) was conducted by packing the samples into an aluminum sample holder. A Rigaku D-Max/400 X-ray diffractometer (Tokyo, Japan) was used. The X-ray beam was nickel-filtered CuKα (λ = 0.154 nm) radiation which was operated at 50 kV. The scanning range was from 1 to 10° and the scanning rate was 2°/min. The corresponding d001 spacings may be calculated by Bragg law. Transmission electron microscopy (TEM) was conducted on a JEM-2010 instrument (JEOL Ltd., Tokyo, Japan). Cryo-ultramicrotomy was used to prepare the ultra-thin films at −100 °C with an acceleration voltage of 80 kV. The thickness of the thin sections was 80–100 nm. Scanning electron microscopy (SEM) was carried out by a Hitachi S-2150 equipment (Hitachi, Tokyo, Japan). The photos were obtained by a potential of 25 kV. The specimens were previously coated with a conductive gold layer.

The curing parameters were measured on an oscillating disk rheometer (MDR-2000, Wuxi, China). The samples were cured at 150 °C under 15 MPa pressure on an electrically heated press. The formulation shown in Table 1 was used to prepare the corresponding samples. The tensile properties were tested using a TCS-2000 universal tensile testing machine (Gotech easting machines company, Taichung, Taiwan) according to Chinese National Standard GB 528-82. These tests were carried out at room temperature with a crosshead speed of 500 mm/min. The samples used were dumbbell and 6 mm wide in cross section. The selected value for each sample was a median value of five specimens. The wear resistant property was performed on a WML-76 Akron abrasion testing machine (Jiangsu Zhenwei Machinery Company, Shanghai, China). In each case, the rotating velocities of the sample and emery wheel were 76 rpm and 33 rpm, respectively, and a pressure of 26.7 N was used on the samples. Shore hardness test was measured by using a XY-1 rubber hardness instrument (Shanghai Chemical Machinery Factory, Shanghai, China).

TGA test was carried out on a Linseis PT-1000 apparatus (Linseis, Selb, Germany). During the test, the atmosphere was nitrogen, and the increasing rate of the temperature was 20 °C/min. The mass of the samples was 10 mg for each test. They were measured in the temperature ranges from room temperature to 600 °C. The flame-retardant property was evaluated using horizontal burning test on a horizontal burning tester (Yang Yi Test Equipment Company, Kunshan, China) according to the standard ASTM D635. In this test, a size of (125 ± 5 mm) × (13.0 ± 0.5 mm) × (3.0 ± 0.2 mm) was used and different rubber sheets were prepared. In addition, the flame-retardant parameters were measured using a Stanton Redcroft cone calorimeter (Stanton Redcroft, London, UK). The heat flux was 25 kW/m^2^ according to ASTM 1356-90. The dimensions of the samples were 10 cm × 10 cm × 2 mm. The cone data reported here were an average of three replicated measurements.

## 3. Results and Discussion

### 3.1. Structure and Properties of FR-DOMt

**Scheme 1:**
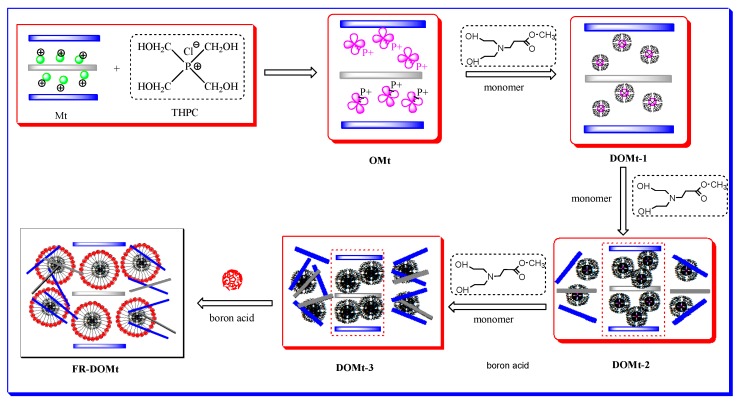
Preparation process of FR-DOMt.

**Figure 1:**
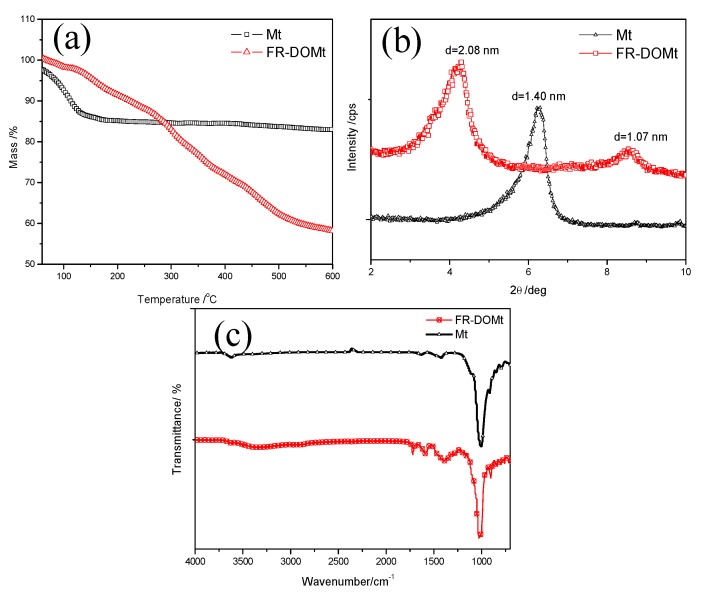

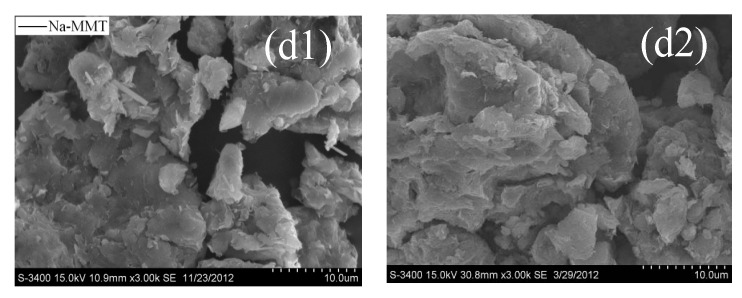
Mt and FR-DOMt (**a**) TGA, (**b**) XRD, (**c**) FTIR, (**d1**,**d2**) SEM.

The preparation process of FR-DOMt was presented in Scheme 1. Firstly, OMt was obtained by a cation exchange reaction between Mt and tetra-hydroxymethyl phosphonium chloride (THPC). Then, three dendrimer modified OMt, DOMt-1, 2, and 3, were prepared from repeated reactions using the momomer, *N*,*N*-dihydroxyl-3-aminomethyl propionate. Lastly, FR-DOMt was fabricated from an esterification reaction between hydroxyl and acid groups [23]. Results exhibited that this FR-DOMt possessed good thermal stability (Figure 1a). It showed improved thermal behavior in the temperature range from 100 to 300 °C. Notably, an increased layered spacing was formed and an exfoliated structure was obtained from the loss of bands from 6.1° and 7.2° (Figure 1b). The new stretching vibrations at 1650 and 1250 cm^−1^ appeared in FTIR spectrum of FR-DOMt (Figure 1c), and these were ascribed to B–O bonds. In addition, the peaks at 3150–3550 cm^−1^ and 1300–1450 cm^−1^ were attributed to N–H groups resulting from the monomer by the dendrimer technology. This analysis together with morphology observation by SEM (Figure 1d1,d2) demonstrated that FR-DOMt possessed a novel structure and composition, and was a new candidate for its application in polymers. 

**Scheme 2:**
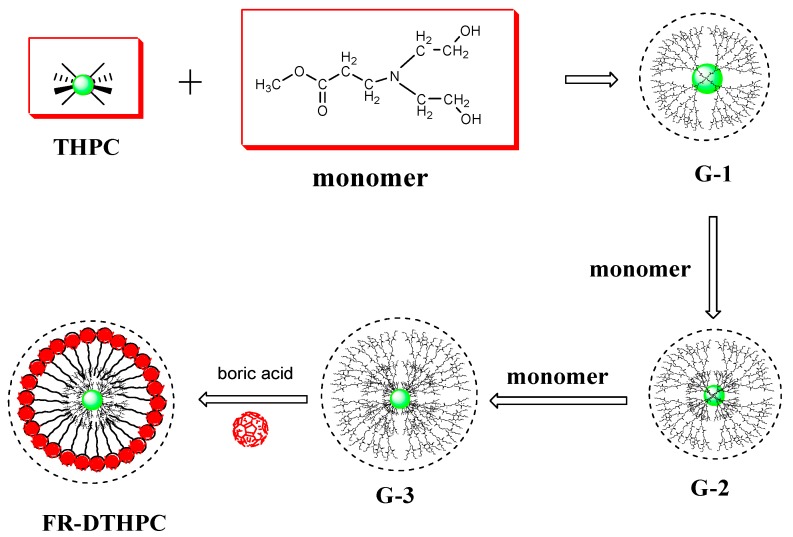
Preparation process of FR-DTHPC.

For a better and easier understanding of synthetic process, the formation of the dendrimer molecules, FR-DTHPC, was given in Scheme 2. In this process, first, THPC was used as the core material, and the monomer, *N*,*N*-dihydroxyl-3-aminmethyl propionate, was used as the branched unit. Then, three generations of dendrimer were synthesized by transesterification. Last, boric acid was used to react with the end groups of G-3, and thus FR-DTHPC was obtained [24]. 

### 3.2. Morphology and Structure of NR/FR-DOMt Nanocomposites

**Figure 2:**
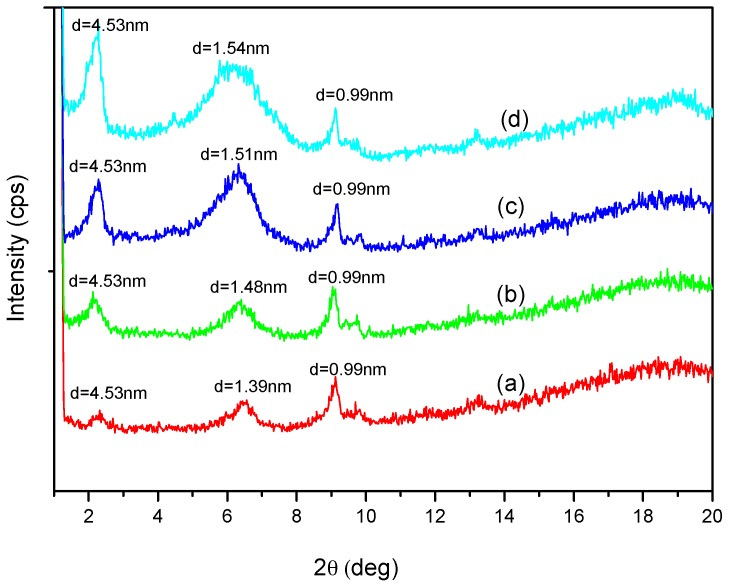
XRD curves of (**a**) NR/FR-DOMt-5, (**b**) NR/FR-DOMt-10, (**c**) NR/FR-DOMt-15, (**d**) NR/FR-DOMt-20.

XRD analysis may be used to characterize the process of intercalation, and thus the intercalated structure of CPN was established. Figure 2 presented XRD curves of NR/FR-DOMt vulcanizates with different amounts of FR-DOMt. All composites exhibited three peaks around 2°, 6°, 9°, and 13°. This was related to the interlayer spacing of 4.5, 1.3, 1, and 0.7 nm, respectively. The two reflections around 2° and 6° (2θ) became more pronounced and broadening. This was resulted from the intercalating of rubber macromolecules into the layered silicates. This also illustrated that the size of organoclay was increased as the clay mineral concentration was increased. Moreover, the peak at about 6° (2θ) shifted toward lower angles for all CPN. This showed the d-spacing was increased due to the intercalation of NR chains into the organoclay structure. The d-spacings of interlayer galleries varied from 1.39 to 1.54 nm. This phenomenon illustrated that the intercalated clay structure was formed in the NR matrix. The dispersion and compatibility of this organoclay were not good due to the more amount of FR-DOMt in the composites. In addition, as illustrated previously, the exfoliated structure was already presented in the organoclay, and thus formation of nanocomposites can be easily obtained during mechanical blending. Therefore, it was envisaged that the reinforcement from intercalated and exfoliated silicate layers and formation of disordered crystal structures were the reasons for following improved mechanical properties of NR nanocomposites.

**Figure 3:**
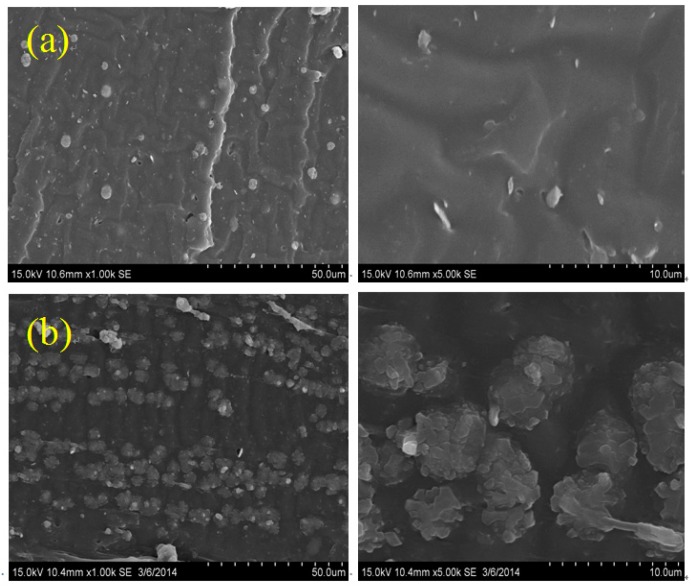

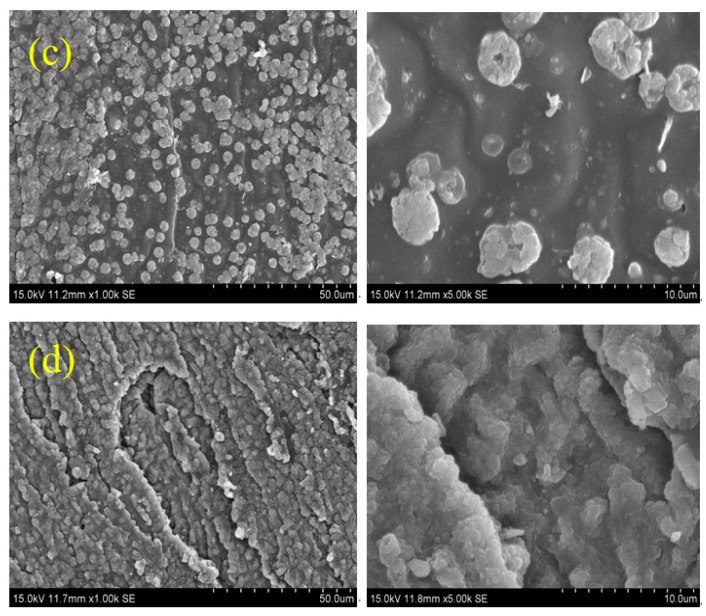
SEM of (**a**) NR/FR-DOMt-5, (**b**) NR/FR-DOMt-10, (**c**) NR/FR-DOMt-15, (**d**) NR/FR-DOMt-20.

SEM was used to investigate morphology of fractured surfaces of NR/FR-DOMt materials. This study was in good agreement with variation of their mechanical properties. The pores in the polymeric matrix may decrease the mechanical properties, while continuous matrix may be endowed with better properties. As expected, with addition of 5 phr of FR-DOMt (Figure 3a), fewer particles were observed. This was resulted from the good compatibility between FR-DOMt and NR matrix [25]. For this blend, crack propagation may be inhibited by the fine dispersion of FR-DOMt together with the altered crack path in NR matrix. In addition, the minor clay phase, FR-DOMt, presented a more uniform distribution in NR/FR-DOMt-10 blend (Figure 3b) [26]. The good compatibility between the components was exhibited from the unidirectional particles in the blend [27]. Based on SEM images of NR/FR-DOMt-15 (Figure 3c), the dendrimer modified silicate layers were loosely dispersed in the polymeric matrix. This phenomenon may be resulted from the shearing force generated during rubber mixing. However, at 20 phr of FR-DOMt, the organoclay was embedded in the NR matrix again and a more compatible morphology was observed (Figure 3d). This was in good accordance with the increased tensile strength of NR/FR-DOMt-20.

**Figure 4:**
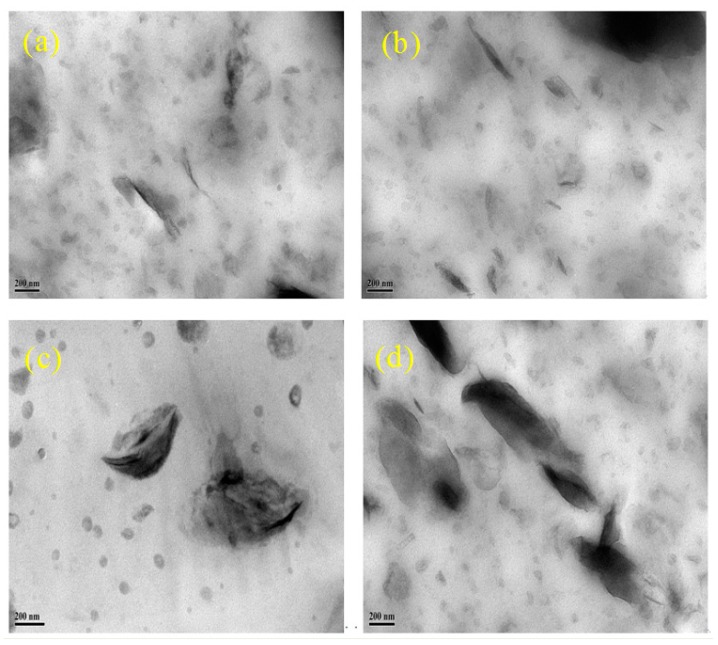
TEM of (**a**) NR/FR-DOMt-5, (**b**) NR/FR-DOMt-10, (**c**) NR/FR-DOMt-15, (**d**) NR/FR-DOMt-20.

**Scheme 3:**
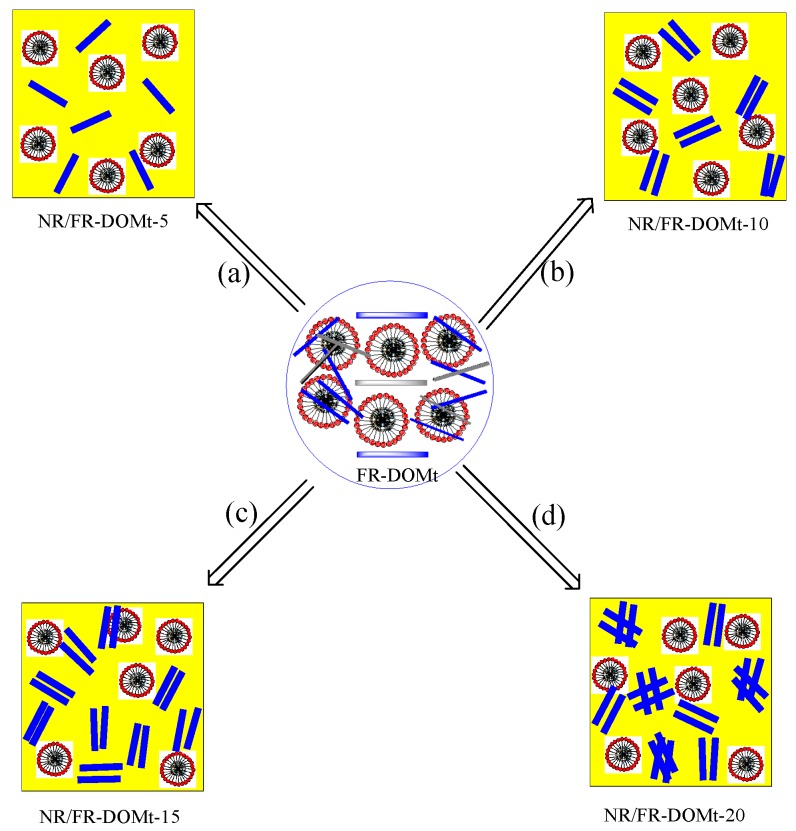
Formation mechanism of (**a**) NR/FR-DOMt-5, (**b**) NR/FR-DOMt-10, (**c**) NR/FR-DOMt-15, (**d**) NR/FR-DOMt-20.

TEM presented further evidence of dispersion state of organoclay in different NR nanocomposites (Figure 4a–d). The formation mechanism of NR/FR-DOMt nanocomposites was proposed in Scheme 3. As can be seen from Figure 4a, the formation of exfoliated structure was almost confirmed in the images of NR nanocomposite based on 5 phr of FR-DOMt. In these images, bright field represented the image of polymer and the dark lines were the cross-section of the layered silicates. In addition, the silicate layers with various distances were observed. After addition of FR-DOMt, the specific interaction from hydrogen bonds between layered silicates and the dendrimer was formed. Thus, randomized exfoliation of silicate layers was inhibited by such an interaction (Scheme 3a) [28]. The relative fraction of intercalation was usually increased by increasing the clay concentration. TEM micrograph of NR/FR-DOMt-10 was shown in Figure 4b. It was shown that the organoclay was heterogeneously dispersed in the NR matrix. In this nanocomposite, partial rubber macromolecules were intercalated into the clay galleries. This was ascribed to the shearing stresses during preparation of the composites (Scheme 3b). However, small tactoids together with a minor aggregated structure exhibited in the NR/FR-DOMt-15 nanocomposite (Figure 4c). These tactoids were constituted by a few silicate layers where the rubber macromolecules were intercalated (Scheme 3c). Moreover, further clay particles agglomeration was generally inevitable to some extent for nanocomposite containing higher clay loading (Figure 4d). It was revealed the coexistence of aggregated and intercalated nanostructures within NR matrix for sample containing 20 phr of nanoclay (Scheme 3d).

### 3.3. Cure Characteristicsof NR/FR-Domt Nanocomposites

**Figure 5:**
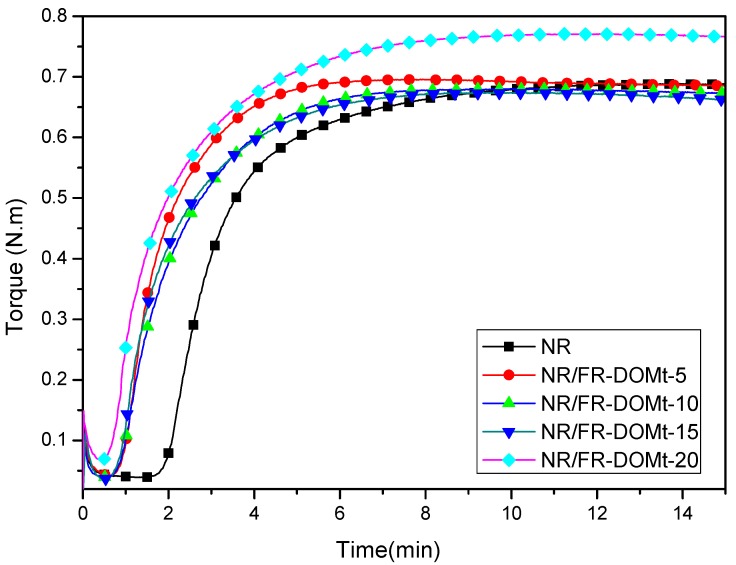
Cure curves of different NR nanocomposites.

The rheographs of different NR nanocomposites at 150 °C were given in Figure 5. Table 2 presented the various curing parameters. From Table 2, the scorch time (t_10_) slowly decreased in the presence of FR-DOMt. This revealed a first accelerated curing process was occurred with addition of FR-DOMt compared to pure NR system. The processing safety of rubber products may be determined from scorch time. The addition of 5~20 phr of FR-DOMt in NR can decrease scorch time and make processing unsafe. The cure time (t_90_) was shorter for the composites than for NR. It was increased with increasing FR-DOMt content. This was ascribed to the presence of boric groups from the ring opening of borate and the presence of dendrimer chains. The boric groups acted as an accelerator and therefore, the vulcanization reaction was brought forward [29]. In addition, the first increase and the later decrease of cure rate values for the nanocomposites also illustrated a first acceleration and a later deceleration of the crosslinking reaction.

For NR/FR-DOMt-20 nanocomposites, the higher viscosity was shown due to the higher minimum torque (M_L_) values, and this led to a decreased material processability. Interestingly, this behavior had no relationship with the clay mineral amount. However, the maximum torque (M_H_) showed some increasing trend with increasing loading of FR-DOMt. Generally, the degree of crosslinking was dependent on the M_H_ obtained during curing process. Assuming M_H_ was related to the crosslinking density, the crosslinking densities of these NR nanocomposites were increased with addition of FR-DOMt.

### 3.4. Mechanical Properties of NR/FR-Domt Nanocomposites

**Figure 6:**
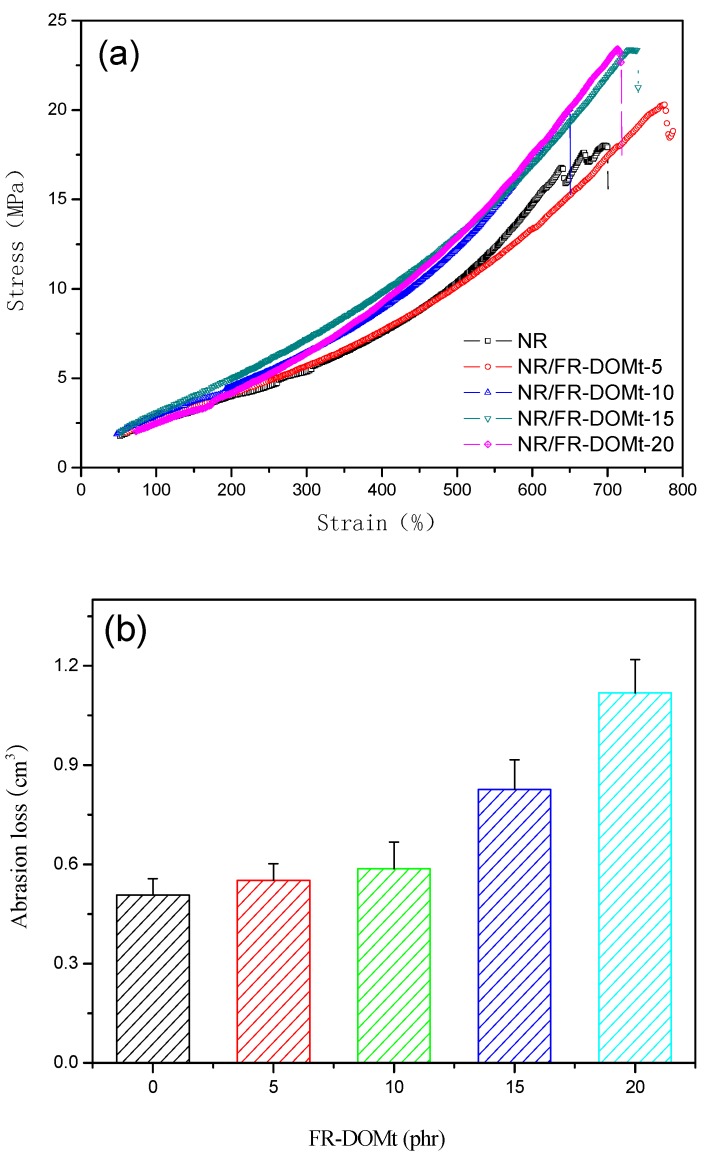

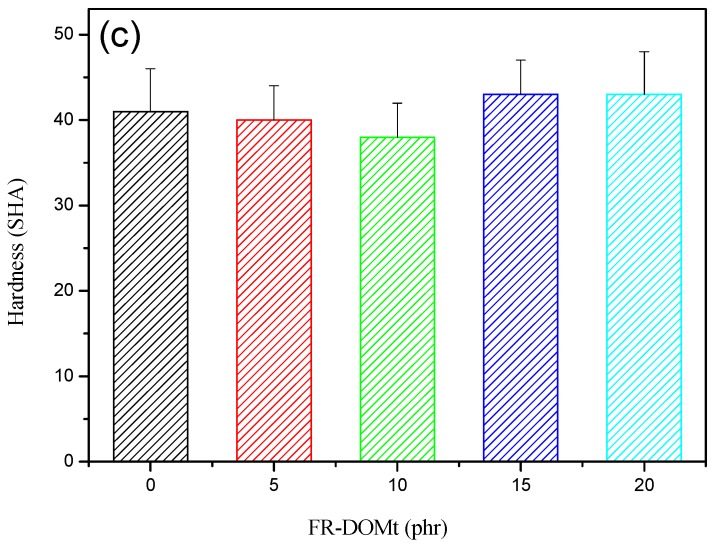
Mechanical properties of different NR nanocomposites (**a**) stress-strain curves, (**b**) abrasion loss, (**c**) hardness.

The mechanical properties of NR/FR-DOMt nanocomposites can be seen from Figure 6. The stress-strain curves showed an increasing trend of slope with the increasing content of FR-DOMt, especially when the elongation was lower than 500% (Figure 6a). The samples with high loading (20 phr) of FR-DOMt exhibited the highest tensile strength. Compared with that of pure NR, 17.5 MPa, the tensile strength of NR/FR-DOMt-20 was increased to 22.2 MPa. The improvement was about 27%. As stated by other authors, the intercalated clays and the dendrimer can provide a larger resistance to mechanical stress [30]. The interactions between layered silicates, the dendrimer, and rubber macromolecular chains were attributed to this increase. The elongation at the break, and ability to material ductility, was increased from 764 to 788% when 5 phr of FR-DOMt was added. Li et al. [29] revealed that sulfur may inhibit the crosslinking of unvulcanized polymeric chains due to the presence of layered silicates. Thus, the tensibility of nanocomposites may be improved, and thus led to the increase of elongation at break. The elongation at break was a little decrease and a later increase with further addition of FR-DOMt. This suggested that no obvious effect on the toughness was exhibited with further loading of this organoclay [31].

In NR with 5~20 phr of FR-DOMt, the loss value presented an obvious decrease with the increasing content of this organoclay (Figure 6b). The NR/FR-DOMt-20 owned an abrasion loss value, 1.12 cm^3^, which was higher than that of the neat NR, 0.51 cm^3^. Abrasion of rubber relied on the resistance of polymer molecular chains to fracture or tearing. The grinding wheel in the Akron machine may give a micro-cutting to the surface of the soft rubber, and this abrasion belonged to the line abrasion mechanism as stated by Burwell [32]. By suppressing tearing of polymers, the abrasion resistance may be improved with addition of reinforcing fillers. However, in this experiment, the abrasion loss was mainly determined by the dendrimer which was easily abraded as filament materials by outside forces. 

The hardness of rubber nanocomposites showed a first decrease and a later increase trend with increasing FR-DOMt content from 5 to 20 phr (Figure 6c). This illustrated that the hardness of the rubber nanocomposites was significantly affected by the clay and dendrimer content. This property was closely related to the strength of the clay and the dendrimer, respectively.

### 3.5. Thermal Properties of NR/FR-Domt Nanocomposites

**Figure 7:**
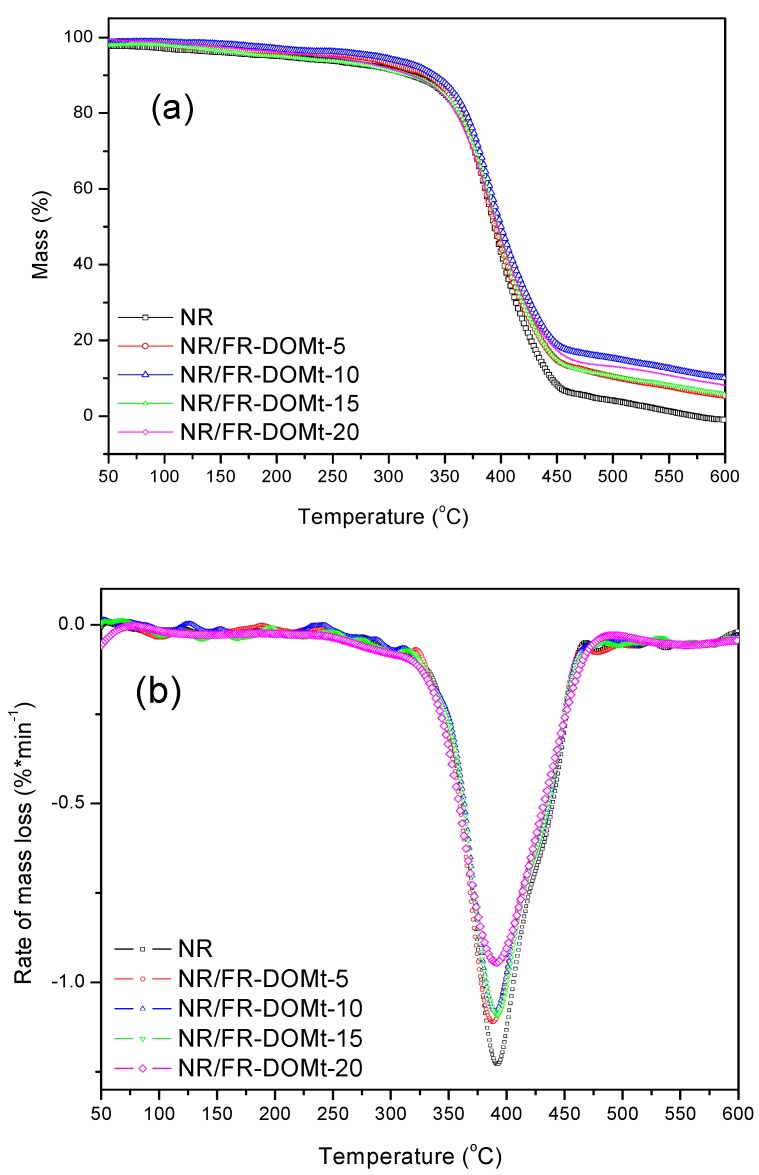
Thermal stability of different NR nanocomposites (**a**) TGA and (**b**) DTG.

TGA and DTG curves of NR nanocomposites were shown in Figure 7a,b, and their corresponding data were summarized in Table 3. From Figure 7a, the thermal degradation of pure NR was made up of one main loss step which was occurred between 370 and 400 °C. All of the other composites with different amounts of FR-DOMt owned similar thermal behavior. The initial thermal degradation temperature (T_5%_) and the residual mass were almost improved after addition of these novel agents. Also, the residual mass in TGA is the highest in sample NR/FR-DOMt-10. It is higher than that of NR/FR-DOMt-20. In this experiment, the thermal stability of the composites may be determined by many factors such as the amount of this filler, the amount of dendrimer and inorganic clay, and pores in the polymeric matrix. In NR/FR-DOMt-20, more pores and more amount of dendrimer may exhibit, and these factors may lead to the easy degradation of this composite. As can be obtained from the maximum decomposition rate and temperature (Figure 7b), these CPNs possessed lower thermal degradation degrees but almost the same temperatures after loading of these particles. This conclusion can also be obtained by other researchers [33,34,35]. In their work, the barrier effect from organoclay may hinder diffusion of small molecules generated during thermal decomposition, and thus can enhance thermal stability of NR nanocomposites. In addition, this phenomenon can be resulted from the lengthened paths of diffused low molecular gases in the polymeric matrix due to the uniform distribution of the layered silicates [17]. However, according to Gao et al. [8], Hoffmann elimination reaction may be occurred due to the presence of alkyl ammonium cations in the organoclay which can help to decompose the rubber matrix. The thermal properties in this temperature range were dominated by this type of reaction.

### 3.6. Flammability Properties of NR/FR-Domt Nanocomposites

Table 4 summarized horizontal burning time and burning rate of different NR/FR-DOMt nanocomposites. Obviously, the burning time of pure NR was relatively shorter (127 s). After addition of 5~20 phr of FR-DOMt, the burning was increased and prolonged. The NR/FR-DOMt-20 possessed a longer burning time, 208 s. The improvement was about 64% due to the flame inhibition and gas barrier effect of FR-DOMt. Correspondingly, these nanocomposites showed a decreased burning rate, from 0.591 to 0.361 mm/s with the addition of FR-DOMt. The decrease was about 39%. In addition, the flammability leve of these novel materials can reach HB grade.

**Figure 8:**
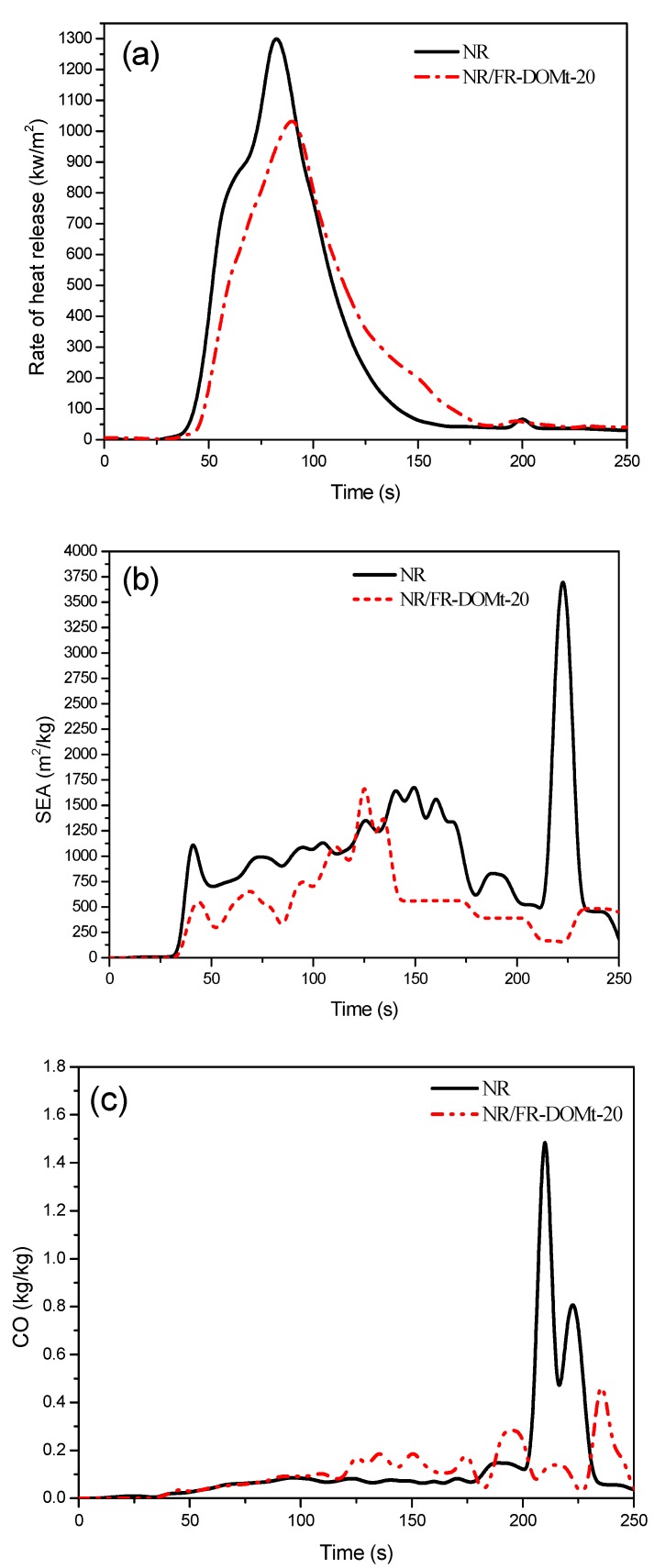
Cone calorimetry curves of NR and NR/FR-DOMt-20 (**a**) HRR, (**b**) SEA, (**c**) CO concentration.

**Scheme 4:**
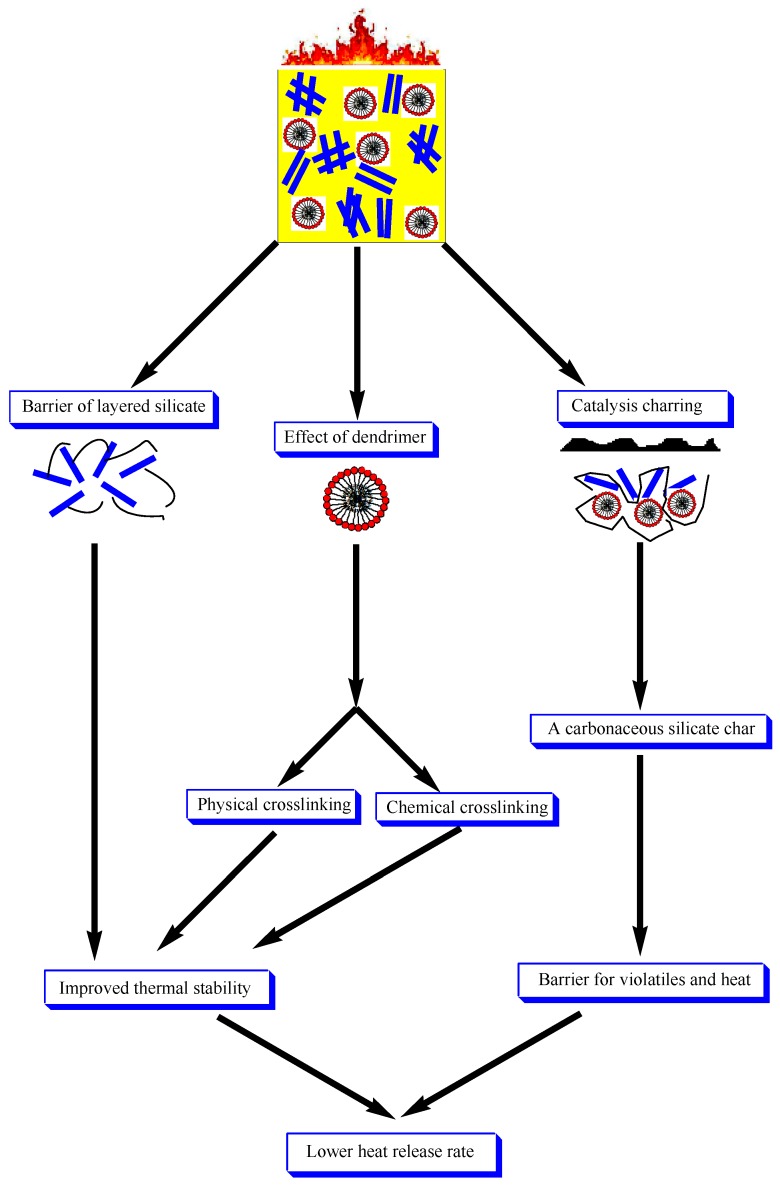
Flame-retardant mechanism of NR/FR-DOMt-20 nanocomposite.

The inhibition to fire of the nanocomposites may be assessed by cone calorimeter test. This test can give a possibility to evaluate burning behavior, smoke formation, and production of toxic gases. Detailed data for NR and its nanocomposite, NR/FR-DOMt-20, from cone calorimeter were reported in Figure 8a–c. Its flame-retardant mechanism was shown in Scheme 4.

The fire hazard of nanocomposites such as fire size and fire growth rate may be measured from heat release rate (HRR). The curves of HRR were shown in Figure 8a. It was revealed that NR burned very quickly after ignition, and a sharp peak HRR (PHRR) of 1319 kW/m^2^ was obtained at 75 s. NR had an obvious single peak, which was due to gradually burning of the samples. The PHRR value of NR/FR-DOMt-20 was 1054 kW/m^2^, which was much lower than that of NR. It can be explained that FR-DOMt decomposed at higher temperatures and char residues were formed on the surface of the samples. These char layers may act as a barrier to prevent heat to the underlying composites and flammable gases into the flame zone during the combustion process. In addition, the dendrimer can improve the crosslinking degree of the nanocomposites. This may improve their thermal stability and the resultant decreased releasing rate of heat.

Detailed information about smoke production can be obtained from SEA test. Figure 8b presented the smoke flux curves of NR and NR/FR-DOMt-20 nanocomposite. Here, at high temperatures, condensed carbon layers may be formed on the surface of NR after addition of FR-DOMt. Moreover, tortuous paths for the flammable small molecules were formed which was credited to the barrier effect of silicates [36]. Thus, smoke production of these composites was significantly reduced.

From Figure 8c, FR-DOMt modified NR produced much lower CO concentration compared with that of pure NR. This was ascribed to a high smoke inhibition ability of boric and silicone elements in this organoclay. Meanwhile, it was observed that CO concentration was obviously increased especially after 210 s. This was resulted from the slow combustion of the char residues, which may be formed by an excellent barrier effect from the organoclay migrated to the surface of the polymeric matrix. Thus, CO may travel along a prolonged and tortuous path due to the barrier effect when volatilizing to its surface.

## 4. Conclusions

NR blends with a novel type of flame-retardant additive, FR-DOMt, were prepared. The impacts of blend ratio and amounts of FR-DOMt on mechanical properties, thermal behavior, and FR properties were studied.

The dispersion status of layered silicates in NR matrix was characterized by XRD, SEM, and TEM. XRD and TEM results revealed that FR-DOMt was intercalated and exfoliated in NR matrix with addition of different amounts of FR-DOMt.

The cure characteristics, mechanical behavior, thermal stability, and flame-retardance of the samples were researched. The experimental data illustrated that the addition of FR-DOMt improved mechanical properties of the samples. In addition, the nanocomposites exhibited higher thermal stability and flame-retardant ability than that of the unfilled NRmatrix.

## Figures and Tables

**Table 1:** Formulation of NR vulcanizates.

Component	Information	phr
NR	3#, industrial grade, provided by Hainan Rubber Company, Hainan, China.	100
sulfur	chemical pure, provided by Shanghai Guoyao Chemical Agent Company, Shanghai, China.	2.5
zinc oxide		5.0
stearic acid		1.0
2-bezothiazolethiol		1.2
tetramethyl thiuram disulfide		0.2
diphenyl guanidine	chemical pure, provided by Shanghai Lingfeng Chemical Agent Company, Shanghai, China.	0.3
antioxidant D	chemical pure, provided by Shanghai Gaoqiao Chemical Agent Company, Shanghai, China.	1.0
FR-DOMt	self-prepared.	0, 5, 10, 15, 20

**Table 2:** Cure characteristics of FR-DOMt filled NR nanocomposites at 150 °C.

Sample	Scorch Time (t_10_) (min)	Cure Time (t_90_) (min)	Minimum Torque (M_L_) (N·m)	Maximum Torque (M_H_) (N·m)	Cure Rate (mm·s^−1^)
NR	2.10	5.75	0.04	0.69	28.30
NR/FR-DOMt-5	1.03	3.60	0.04	0.70	40.23
NR/FR-DOMt-10	1.02	4.32	0.04	0.68	31.09
NR/FR-DOMt-15	0.95	4.35	0.04	0.67	30.15
NR/FR-DOMt-20	0.80	4.73	0.07	0.77	25.75

**Table 3:** TGA data of different NR nanocomposites.

Materials	T_5%_ (°C)	T_10%_ (°C)	T_max_ (°C)	Residual Mass (%)
NR	282.7	342.5	393.7	1.57
NR/FR-DOMt-5	284.1	343.6	392.0	4.97
NR/FR-DOMt-10	294.5	345.9	391.4	10.29
NR/FR-DOMt-15	281.7	344.0	393.6	5.18
NR/FR-DOMt-20	287.6	338.4	393.9	7.85

**Table 4:** Horizontal burning (HB) results of different NR nanocomposites.

Materials	Horizontal Burning Test		Burning Rate (mm·s^−1^)
	Burning Distance (mm)	Burning Time (s)	
NR	75	127	0.591
NR/FR-DOMt-5	75	133	0.564
NR/FR-DOMt-10	75	190	0.395
NR/FR-DOMt-15	75	192	0.391
NR/FR-DOMt-20	75	208	0.361
